# ZrB_2_-CNTs Nanocomposites Fabricated by Spark Plasma Sintering

**DOI:** 10.3390/ma9120967

**Published:** 2016-11-29

**Authors:** Hua Jin, Songhe Meng, Weihua Xie, Chenghai Xu, Jiahong Niu

**Affiliations:** National Key Laboratory of Science and Technology on Advanced Composites in Special Environments, Harbin Institute of Technology, Harbin 150001, China; 2007hit@163.com (H.J.); mengsh@hit.edu.cn (S.M.); hit-xuchenghai@163.com (C.X.); niujhhit@126.com (J.N.)

**Keywords:** nanocomposites, carbon nanotubes (CNTs), ZrB_2_, fracture toughness, strength

## Abstract

ZrB_2_-based nanocomposites with and without carbon nanotubes (CNTs) as reinforcement were prepared at 1600 °C by spark plasma sintering. The effects of CNTs on the microstructure and mechanical properties of nano-ZrB_2_ matrix composites were studied. The results indicated that adding CNTs can inhibit the abnormal grain growth of ZrB_2_ grains and improve the fracture toughness of the composites. The toughness mechanisms were crack deflection, crack bridging, debonding, and pull-out of CNTs. The experimental results of the nanograined ZrB_2_-CNTs composites were compared with those of the micro-grained ZrB_2_-CNTs composites. Due to the small size and surface effects, the nanograined ZrB_2_-CNTs composites exhibited stronger mechanical properties: the hardness, flexural strength and fracture toughness were 18.7 ± 0.2 GPa, 1016 ± 75 MPa, and 8.5 ± 0.4 MPa·m^1/2^, respectively.

## 1. Introduction

Zirconium diboride (ZrB_2_) displays a number of attractive properties, such as low density, good chemical stability, high melting point, hardness, and thermal and electrical conductivity [1,2,3], which are desirable for structural applications like cutting tools, refractory materials in foundries, electrical devices, nozzles, and armor. The major problem regarding the sintering behavior of ZrB_2_ is the nature of barriers to densification due to the strong covalent bonding, low self-diffusion, and presence of oxide on the surface of particles [4,5,6]. Earlier studies have shown that reduction of the starting particle size and the use of sintering aids can effectively enhance the densification [7,8]. In addition, it is believed that miniaturizing the grain size to a nanoscale level can enable the creation of nanograined ceramics that could greatly improve its mechanical properties [9,10,11]. With technological advancements in powder preparation and the emergence of nanoscale materials, some researchers have fabricated ZrB_2_-based composites by introducing nanosized ceramic particles into the ceramic-matrix grains or grain boundaries [12,13]. The most significant achievements with this approach have been obtained by Guo and Liu, who reported that the introduction of nanosized SiC particles into ZrB_2_ increased the strength of the composites [14,15]. However, despite having huge potential, no commercial sources for the preparation of nanograined ZrB_2_ ceramics have been developed in the last decades.

To prepare nanostructured ZrB_2_-based ceramics, nanoscale powder and rapid sintering processes are required to inhibit abnormal grain growth during the sintering process. In this respect, the conventional sintering techniques (hot pressing, pressureless sintering, etc.) are quite challenging, due to the high temperatures and long dwelling times involved that lead to considerable grain coarsening in the product [16,17]. By employing a pulsed direct current (DC) current to improve sintering kinetics, spark plasma sintering (SPS) has emerged as a promising approach for densifying a number of poorly sinterable ceramics at a lower temperature and in a shorter time, while preserving an ultra-fine grain size [18,19,20]. Previous studies on ZrB_2_-based ceramic materials showed that SPS enhanced densification and refined the microstructure in very short processing cycles [21,22]. However, the unsatisfactory value of the toughness still is an obstacle for the wide use of ZrB_2_-based ceramics, especially for applications in severe environments. Accordingly, properties must be improved before the potential ZrB_2_ applications can be fully realized. There exist two methods for toughening ceramics: one is to avoid the occurrence of crack sources, and the other is to introduce a second phase with toughening capabilities, such as particles, whiskers, and fibers. For example, Sun et al. [23] reported that the fracture toughness is increased from 3.5 MPa·m^1/2^ for pure ZrB_2_ to 7.1 MPa·m^1/2^ for ZrB_2_ with 40 vol % Nb. Zhu et al. [24] prepared ZrB_2_-based ceramics with 20 vol % SiC whiskers by hot-pressing at 1800 °C, and produced composites that showed a high fracture toughness of 6.7 MPa·m^1/2^. Lin et al. [25] fabricated fully dense ceramics of ZrO_2_ fibers with ZrB_2_ matrix sintered at 1950 °C by hot-pressing; the flexural strength and fracture toughness reached 633 MPa and 5.6 MPa·m^1/2^, respectively.

Since their discovery, carbon nanotubes (CNTs) have emerged as potentially attractive reinforcing materials in composites—particularly in ceramic—matrix composites—due to their exceptional mechanical and physical properties [26,27]. Yavas et al. [28] reported on B_4_C matrix composites with good properties that were reinforced by CNTs. Sha et al. [29] found that ZrC-based ceramic composites doped with 20 vol % Ti and 3 vol % CNTs had higher strength and toughness, compared with the monolithic ZrC. Meanwhile, Saheb et al. [30] reinforced Al_2_O_3_ by SiC and CNTs using a combination of ball milling, sonication, and SPS. The authors reported fracture toughness values of up to 6.9 MPa·m^1/2^ for the composites compared with the value of 3.5 MPa·m^1/2^ for the monolithic alumina. To date, there have been fewer reports on CNTs toughening ZrB_2_-based ceramics. Tian and Lin et al. [31,32] reported that the addition of CNTs produced promising results. For instance, for ZrB_2_-based composites, fracture toughness values up to 5.6–7.2 MPa·m^1/2^ were reported.

In this paper, CNTs were chosen as the reinforcement. Nanograined ZrB_2_ ceramics with and without the addition of CNTs were fabricated by SPS. For comparison, coarse-grained ZrB_2_-CNTs composites were also sintered by SPS. The microstructure and mechanical properties of the composites were investigated.

## 2. Experimental Procedures

**Figure 1:**
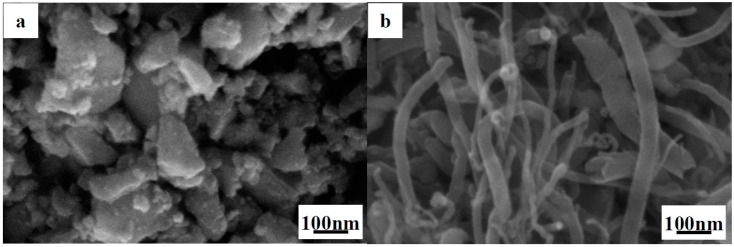
Micrographs of the as-received powders by secondary electron scanning electron microscopy (SEM): (**a**) nano-ZrB_2_ and (**b**) multi-walled carbon nanotubes (CNTs).

This study was conducted using commercially available powders of nano-ZrB_2_ (60 nm, >95%, Kaier Nanometer Energy & Technology Co. Ltd., Hefei, China), micro-ZrB_2_ (1–2 μm, >99.5%, Northwest Institute for Nonferrous Metal Research, Xi’an China), and multi-walled CNTs (Mean diameter and length are 40–60 nm and 5–15 μm, respectively, >99.9%, Shenzhen Nanotech Port Co. Ltd., Shenzhen, China). The weights of the powders used were in proportion to the stoichiometric ratio to yield ZrB_2(nano)_-*x* wt % CNTs (*x* = 0, 1, 3, 5, 7, and 10). Scanning electron microscopy (SEM) images of the nano-ZrB_2_ and CNTs powders are presented in Figure 1. Before mixing, the nano-ZrB_2_ and CNTs were first dispersed, separately, by ultra-sonication and mechanical homogenization for 1 h using polyethylene imine (PEI, MW 10,000) as a dispersant and ethanol as a solvent. Then, the nano-ZrB_2_ and CNTs suspensions were mixed in a ball mill, with ZrO_2_ balls as the ball milling media at 220 rpm for 4 h. After drying in a rotating evaporator, the powder mixtures were sintered by SPS under vacuum at 1600 °C for 10 min under a uniaxial load of 30 MPa using an inductively heated graphite die lined with a BN-coated graphitized sheet. For comparison, the ZrB_2(micro)_-*y* wt % CNTs (*y* = 0, 1, 3, 5, 7, and 10) composite was sintered under the same conditions.

After sintering, the surfaces of the samples were ground to remove the graphite layer, and were then polished with 1 μm diamond slurry. The bulk density of the consolidated specimens was measured using the Archimedes method with deionized water as the immersing medium. The theoretical densities of the specimens were calculated according to the rule of mixtures, using 6.09 g·cm^3^ and 1.8 g·cm^3^ as theoretical densities for ZrB_2_ and CNTs, respectively. The relative density was calculated by dividing the bulk density by the theoretical density. Phases were identified by conventional X-ray diffraction (XRD; PANalytical X’Pert PRO, Holland, The Netherlands, CuKα = 1.5418 Å). Microstructural observation was conducted by SEM (ZEISS EVO18, Carl Zeiss Microscopy GmbH, Goettingen, Germany).

Hardness was evaluated by Vickers’ indentation with a 50 N load applied on the polished sections for 10 s. The bending strength was assessed by a three-point bending test, using a 12 mm span and a crosshead speed of 0.5 mm·min^−1^. Test samples were machined into bars of 2 mm × 3 mm × 18 mm (width × height × length) and polished with diamond slurries down to a 1 μm finish. The edges of all the specimens were chamfered to minimize the effect of stress concentration due to machining flaws. Fracture toughness (*K*_IC_) was evaluated by a single-edge notched beam test with a 16-mm span and a crosshead speed of 0.05 mm·min^−1^, on the same jig used for the flexural strength. The test bars—2 mm × 4 mm × 22 mm (width × height × length)—were notched with a 0.1 mm-thick diamond saw, and the notch length was about half the height of the bar.

## 3. Results and Discussion

**Figure 2:**
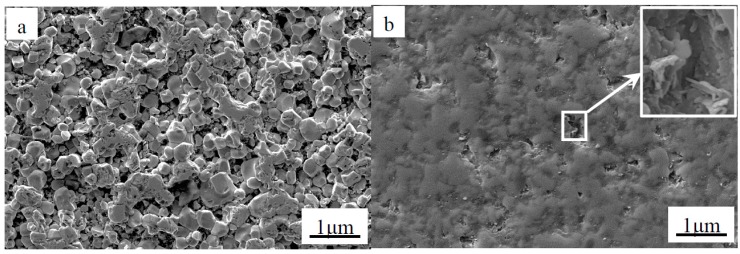
Secondary electron SEM images of the polished surface of as-sintered (**a**) monolithic ZrB_2(nano)_ and (**b**) ZrB_2(nano)_-5 wt % CNTs composite.

The characteristics of the ZrB_2(nano)_-*x* wt % CNTs composites are listed in Table 1. The single-phase ZrB_2(nano)_ ceramic sample had a relative density of 80.9%. The addition of CNTs to composites had obvious effects on the density of the final product. Figure 2 shows the SEM images of the polished surface of as-sintered monolithic ZrB_2(nano)_ and ZrB_2(nano)_-5 wt % CNTs composite. Obvious open porosity could be found in monolithic ZrB_2(nano)_ (Figure 2a), and the surface of monolithic ZrB_2(nano)_ was difficult to be polished due to the lower relative density. Compared to monolithic ZrB_2(nano)_, nanocomposites containing CNTs had higher relative density values and achieved near full density (98.2%) when the CNT content was 5 wt %. The microstructure of the polished surface of ZrB_2(nano)_-5 wt % CNTs composite is presented in Figure 2b. As shown in the high magnifications in Figure 2b, the porosity was most likely caused by the removal of CNTs during polishing. When the CNTs content was up to 7 wt %, the relative density was slightly decreased. In this comparison, it was assumed that the relative density of the composites was related to the dispersion of CNTs, and the shape and size of the mixture particles, which will be discussed later. For comparison, the characteristics of the ZrB_2(micro)_-*y* wt % CNTs composites are listed in Table 2. Likewise, the relative density of these composites reached the peak value of 90.1% when the CNTs content was 5 wt %, and then decreased. Additionally, it was noted that the relative density had a higher value when the ZrB_2_ diameter decreased from microscale to nanoscale, as shown in Table 1 and Table 2. As is well known, monolithic ZrB_2_ has poor sinterability, and obtaining fully dense ceramic is difficult. In this paper, the sintering temperature was only 1600 °C, and the dwelling time was 10 min, but the high relative density of the composites was obtained due to the use of SPS. In this process, the spark impact pressure, Joule heating, and an electrical field diffusion effect could be generated by the DC pulse discharge, so the ZrB_2_–based composites could be rapidly sintered under a relatively lower temperature and short period of time.

The mechanical properties of the ZrB_2(nano)_-*x* wt % CNTs and ZrB_2(micro)_-*y* wt % CNTs composites are also listed in Table 1 and Table 2. The results revealed that the hardness, flexural strength, and fracture toughness of the ZrB_2(nano)_-CNTs composites were higher than those of similar ZrB_2(micro)_-CNTs composites, and increased as the CNTs content was increased from 0 to 5 wt %, but then decreased as the CNTs content increased from 7 wt % to 10 wt %. As established, the mechanical properties of a material are generally decreased by the introduction of weak second phases, such as pores. Actually, it was understood that the increased mechanical properties resulted from the enhanced densification. On the other hand, the fine grains and the dispersion of the CNTs were considered the other dominant factors in the improvement of the mechanical properties of the material. The reduced grain size increased the number of crack deflections and total fracture paths. Consequently, the crack extension and deflection consumed more fracture energy, leading to higher hardness, strength, and fracture toughness.

**Figure 3:**
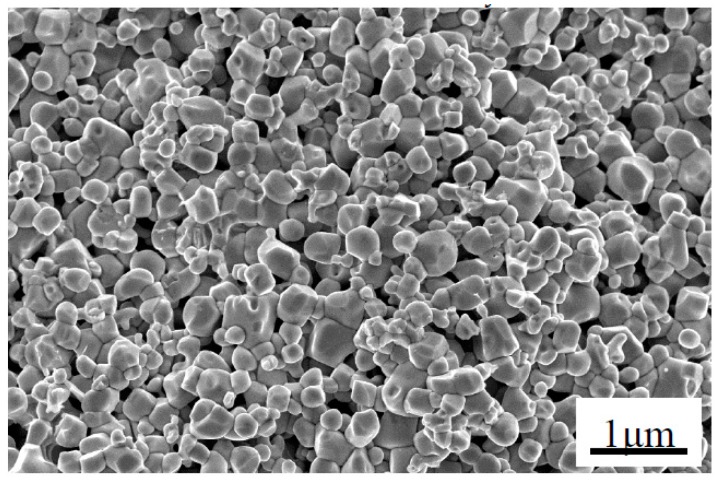
Secondary electron SEM images of fracture surface of monolithic ZrB_2(nano)_.

**Figure 4:**
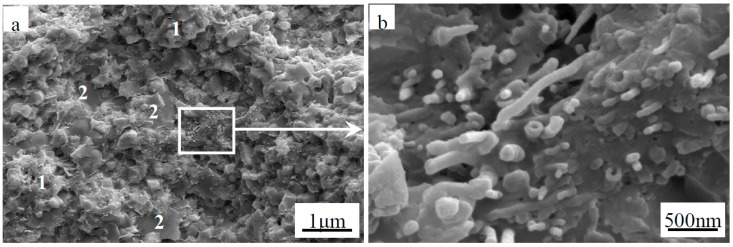
Secondary electron SEM images of fracture surface of (**a**) ZrB_2(nano)_-5 wt % CNTs composites (“1” represents intergranular fracture, “2” represents transgranular fracture); and (**b**) magnified region.

**Figure 5:**
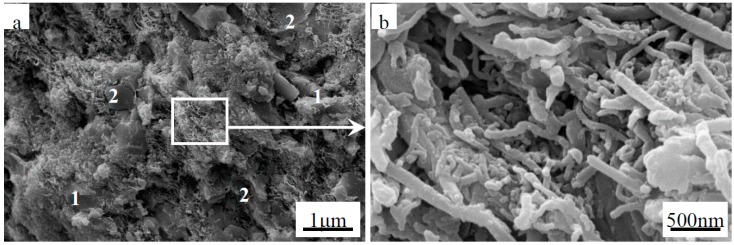
Secondary electron SEM images of (**a**) fracture surface of ZrB_2(nano)_-7 wt % CNTs composites (“1” represents intergranular fracture, “2” represents transgranular fracture); and (**b**) magnified region.

**Figure 6:**
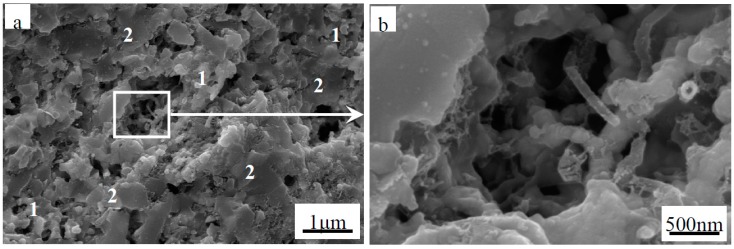
Secondary electron SEM images of (**a**) fracture surface of ZrB_2(micro)_-5 wt % CNTs composites (“1” represents intergranular fracture, “2” represents transgranular fracture); and (**b**) magnified region.

The fracture surface of monolithic ZrB_2(nano)_ sintered at 1600 °C by SPS was observed by SEM, as shown in Figure 3. Many pores were present in the monolithic ZrB_2(nano)_ ceramic, and the ZrB_2(nano)_ grains coarsened significantly, which is in agreement with the relative density data presented in Table 1. Compared with the monolithic ZrB_2(nano)_ ceramic, the achievement of refined and pore-free microstructures presented in Figure 4 highlights the beneficial role that CNTs played in preventing the coalescence of ZrB_2_ grains and improving the densification of refractory matrices. When the amount of CNTs was increased to 7 wt % or more (Figure 5), a porous rope-like structure of CNTs clusters was observed (indicated in the high magnifications in Figure 5). This structure resulted in the slight reduction of the relative density, which was consistent with the result of the relative density analysis. Additionally, this porous rope-like structure of the CNTs clusters was not advantageous for the improvement of the reinforcing effect of CNTs, which led to the reduction of the mechanical properties of the material, as shown in Table 1 and Table 2. The SEM micrographs of the fracture surfaces of the ZrB_2(micro)_-5 wt % CNTs composite are shown in Figure 6, where the presence of a few pores was evident in the ZrB_2(micro)_-CNTs composite, consistent with the rather low relative density. Grain growth in the ZrB_2(nano)_-CNTs and ZrB_2(micro)_-CNTs composites occurred by the same basic mechanism. The CNTs distributed at the interface of the ZrB_2_ grains and prevented the ZrB_2_ grain boundaries from moving by pinning the boundaries, so that the grain growth is clearly hindered. However, compared with the micro-sized ZrB_2(micro)_-CNTs composite, the fully dense ZrB_2(nano)_-CNTs nanocomposite could be obtained at such low sintering temperature due to the small-size effect and surface effect based on the nanometer theory.

**Figure 7:**
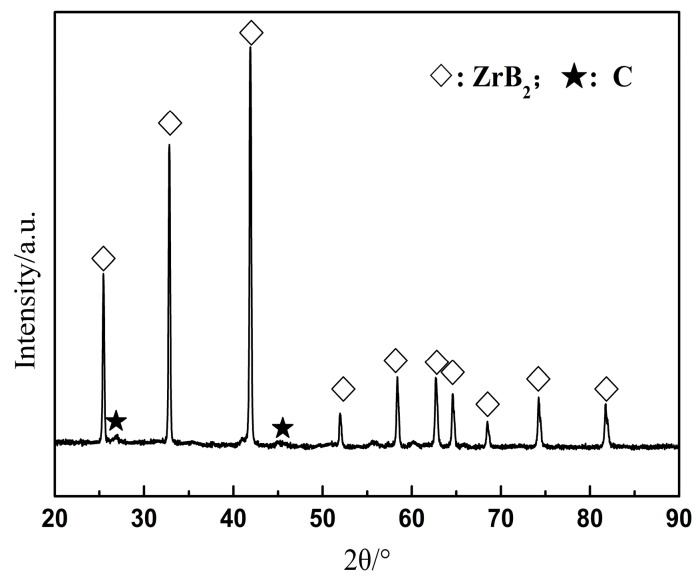
X-ray diffraction (XRD) patterns of ZrB_2(nano)_-5 wt % CNTs composites sintered at 1600 °C.

**Figure 8:**
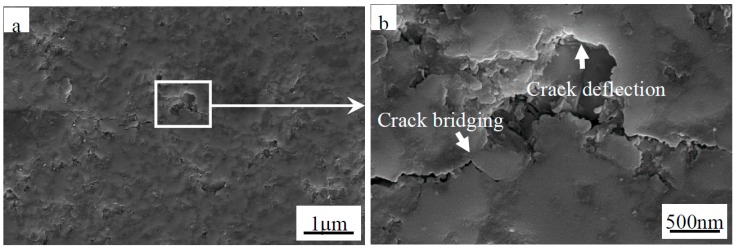
Secondary electron SEM images showing (**a**) an indentation crack for the ZrB_2(nano)_-5 wt % CNTs composites; and (**b**) magnified region.

Meanwhile, the primary fracture mode of the monolithic ZrB_2(nano)_, ZrB_2(nano)_–CNTs and ZrB_2(micro)_-CNTs composites can be observed in Figure 3, Figure 4, Figure 5 and Figure 6. In particular, for the monolithic ZrB_2(nano)_, the fracture mode was mainly intergranular, but ZrB_2(nano)_-CNTs and ZrB_2(micro)_-CNTs showed a mixed of transgranular and intergranular fracture mode (as marked in Figure 4, Figure 5 and Figure 6). The analysis of the microstructure at the high magnifications shown in Figure 4b and Figure 6b revealed the perfect interface between CNTs and the ZrB_2_ matrix—confirmed by the XRD analysis, which showed the absence of any obvious reaction between CNTs and ZrB_2_ (Figure 7). The significant CNTs roots herein demonstrated the debonding and pull-out of CNTs during the fracture process. On the other hand, the rough fracture surface of the ceramics and the ragged crack propagation path indicated the appearance of crack deflection. In order to appreciate the effect of CNTs on the crack propagation models, the typical crack propagation paths obtained by Vickers’ indentation are presented in Figure 8. The tortuous crack propagation path indicated that crack deflection occurred along the weak interface. Additionally, evident crack bridging was displayed. Moreover, as it is known, when a relatively brittle matrix is enhanced by reinforcement (like the CNTs used in this work), debonding of the CNTs can be found around the weak interface of the CNTs and the matrix. Therefore, intact CNTs can be found when the crack propagates around them and the length and area of the opening cracks are increased, resulting in crack deflection and bridging, which decrease the stress intensity around the crack tip. Furthermore, when the crack finally propagates, significant fracture energy is gained from frictional sliding during the CNTs pullout. Thus, compared with the monolithic ZrB_2_, adding CNTs to the ZrB_2_ matrix can improve the fracture toughness (as shown in Table 1), and in the ZrB_2(nano)_-CNTs composite, it had a maximum value of 8.5 ± 0.4 MPa·m^1/2^. However, for the ZrB_2(micro)_-CNTs composite, the value of the fracture toughness was only 6.9 ± 0.3 MPa·m^1/2^ in the site of 90.1% relative density. This was attributed to the creation of more grain boundaries by the leading fine grains, which can prevent the propagation of cracks by the toughening mechanism of crack deflection and expend much more fracture energy. Moreover, the ZrB_2(nano)_-CNTs composite had the highest flexural strength and hardness (1016 ± 75 MPa and 18.7 ± 0.2 GPa, respectively), which were much higher than those of the monolithic ZrB_2(nano)_ and ZrB_2(micro)_-CNTs composites. This was mainly due to the high densification and fine microstructures obtained in the ZrB_2(nano)_-CNTs composites.

## 4. Conclusions

ZrB_2_-based materials were fabricated by SPS with and without CNTs as reinforcement. The effect of the CNT content on the microstructure and mechanical properties of the ZrB_2_-CNTs sintered by SPS was also investigated. The SPS technique allowed dense materials to be produced at a lower temperature and in shorter time, without the addition of sintering aids. Compared with the ZrB_2(micro)_-CNTs composites, the highest hardness (18.7 ± 0.2 GPa), flexural strength (1016 ± 75 MPa), and fracture toughness (8.5 ± 0.4 MPa·m^1/2^) were achieved in ZrB_2(nano)_-CNTs composites sintered at 1600 °C, which was attributed to the high densification and fine microstructures obtained in ZrB_2(nano)_-CNTs composites. The effect of the addition of CNTs on the microstructure and mechanical properties of composites was also studied. The results showed that the addition of CNTs can inhibit the abnormal growth of ZrB_2_ grains and improve the fracture toughness of the composites. The toughness mechanisms were crack deflection, crack bridging, and debonding and pull out of CNTs.

## Figures and Tables

**Table 1:** Density and mechanical properties of the ZrB_2(nano)_-*x* wt % carbon nanotubes (CNTs) composites.

Material	Relative Density (%)	Hardness (GPa)	Flexural Strength (MPa)	Fracture Toughness (MPa·m^1/2^)
Pure ZrB_2(nano)_	80.9 ± 0.5	14.8 ± 0.4	595 ± 62	4.1 ± 0.2
ZrB_2(nano)_-1 wt % CNTs	92.6 ± 0.4	17.8 ± 0.2	789 ± 72	6.4 ± 0.3
ZrB_2(nano)_-3 wt % CNTs	97.2 ± 0.2	18.2 ± 0.3	902 ± 56	7.8 ± 0.2
ZrB_2(nano)_-5 wt % CNTs	98.2 ± 0.3	18.7 ± 0.2	1016 ± 75	8.5 ± 0.4
ZrB_2(nano)_-7 wt % CNTs	98.0 ± 0.5	18.6 ± 0.3	985 ± 64	8.2 ± 0.3
ZrB_2(nano)_-10 wt % CNTs	97.9 ± 0.4	18.5 ± 0.4	964 ± 48	8.1 ± 0.2

**Table 2:** Density and mechanical properties of ZrB_2(micro)_-*y* wt % CNTs composites.

Material	Relative Density (%)	Hardness (GPa)	Flexural Strength (MPa)	Fracture Toughness (MPa·m^1/2^)
Pure ZrB_2(micro)_	74.2 ± 0.6	12.6 ± 0.3	324 ± 35	3.4 ± 0.2
ZrB_2(micro)_-1 wt % CNTs	80.6 ± 0.2	15.7 ± 0.4	512 ± 46	5.7 ± 0.4
ZrB_2(micro)_-3 wt % CNTs	87.4 ± 0.4	16.0 ± 0.3	597 ± 52	6.3 ± 0.3
ZrB_2(micro)_-5 wt % CNTs	90.1 ± 0.3	17.4 ± 0.1	638 ± 50	6.9 ± 0.3
ZrB_2(micro)_-7 wt % CNTs	90.0 ± 0.5	17.2 ± 0.2	622 ± 64	6.8 ± 0.2
ZrB_2(micro)_-10 wt % CNTs	89.8 ± 0.3	16.9 ± 0.2	615 ± 45	6.6 ± 0.1
